# Supplementary material for: MALDI-TOF MS and 16S RNA Identification of Culturable Gastric Microbiota: Variability Associated with the Presence of Helicobacter pylori
Source: Microorganisms. 2020 Nov 10;8(11):1763. doi: 10.3390/microorganisms8111763 (PMC7697671; doi:10.3390/microorganisms8111763)
Supplement: Supplementary file 1 [file microorganisms-08-01763-s001.pdf]

microorganisms

MDPI

Article

MALDI-TOF MS and 16S RNA Identification of Culturable Gastric Microbiota: Variability associated with the Presence of *Helicobacter pylori*

Claudia Troncoso [1,2], Mónica Pavez [3,*], Álvaro Cerda [4], Marcelo Oporto [3], Daniel Villarroel [3], Edmundo Hofmann [5,6,7], Eddy Ríos [5,6,7], Armando Sierralta [5,6,7], Luis Copelli [8] and Leticia Barrientos [2,*]

1. Programa de Doctorado en Ciencias, Mención Biología Celular y Molecular Aplicada, Universidad de La Frontera, 01145, Temuco, Chile; troncosomunozc@gmail.com
2. Facultad de Ciencias de la Salud, Universidad Autónoma de Chile, 01090, Temuco, Chile
3. Laboratorio de Biología Molecular Aplicada, Centro de Excelencia en Medicina Traslacional, Núcleo Científico y Tecnológico en Biorecursos (BIOREN), Universidad de La Frontera, 01145, Temuco, Chile; m.oporto01@ufromail.cl (M.O.); d.villarroel02@ufromail.cl (D.V.)
4. Laboratorio de Bioanálisis Molecular. Centro de Excelencia en Medicina Traslacional, Universidad de La Frontera, 01145, Temuco, Chile; alvaro.cerda@ufrontera.cl
5. Departamento de Medicina Interna, Facultad de Medicina, Universidad de La Frontera, 01145, Temuco, Chile; edmundo.hofmann@ufrontera.cl (E.H.); eddy.rios@ufrontera.cl (E.R.); armando.sierralta@gmail.com (A.S.)
6. Unidad de Gastroenterología, Hospital Hernán Henríquez Aravena, 4781151, Temuco, Chile
7. Unidad de Gastroenterología, Clínica Alemana de Temuco, Temuco, Chile
8. Unidad de Gastroenterología, Hospital de Villarrica, Villarrica, Chile; luis.coppelli@gmail.com

* Correspondence: leticia.barrientos@ufrontera.cl (L.B.); monica.pavez@ufrontera.cl (M.P.); Tel.: +56-45-2592802

Received: 25 August 2020; Accepted: 24 September 2020; Published: date

Abstract: *Helicobacter pylori* is the main bacteria associated with gastroduodenal diseases. Recent studies have reported that gastric microbiota might be modified by the *H. pylori* colonization, favoring gastric lesions' development. In Chile, the region of La Araucanía concentrates a high risk of gastric cancer associated with *Helicobacter pylori* colonization, rurality, poverty, and Mapuche ethnicity. Hence, we aimed to identify the culturable gastric microbiota and characterize its variability at different stages of epithelial injury, based on its *H. pylori* colonization in dyspeptic patients from this Chilean region. Microaerophilic bacteria strains were isolated from antrum biopsies of 155 dyspeptic patients' biopsies and identified using MALDI-TOF MS or *16sRNA* gene sequencing for non-pylori species identification, and *UreC* gene amplification for *H. pylori* confirmation. We found 48 species from 18 families, mainly belonging to *Neisseriaceae* (21.3%), *Streptococcaceae* (20.0%), *Actynomicetaceae* (9.0%), *Enterobacteriaceae*, and *Lactobacillaceae* (4.5%); however, *Streptococcaceae* and *Actinomycetaceae* families showed a significant reduction in samples infected with *H. pylori*, along with a considerably lower diversity of species. Our results revealed a microbiota modification due to *H. pylori* colonization associated with the gastric epithelial state, suggesting a potential microbiota role for developing and progressing gastric diseases.

Keywords: *Helicobacter pylori*; Gastric lesion; microaerophilic bacteria; gastric microbiota

1. Introduction

Commented [M1]: Please proof on this version as we have already done the English editing and formatting changes. Please do the proofreading with track changes and do not delete the comments. Thank you.
Please carefully check the accuracy of the title. Please check through and make sure everything is fine and no content missing.

Commented [M2]: Please carefully check the accuracy of names and affiliations. Changes will not be possible after proofreading.

Commented [M3]: M ónica Pavez is different from system, please confirm.

Commented [M4]: Daniel Villarroel is different from system, please confirm.

Commented [M5]: Eddy R ós is different from system, please confirm.

Commented [M6]: Is this postcode right? Please check the postcode throughout the affiliation with yellow highlight.

Commented [M7]: Affiliation number should be used in numerical order. 2 should be after 1. Please confirm.

Commented [M8]: Please confirm is it right?

Commented [M9]: Please add the postcode.

Commented [M10]: Please add the postcode.

Commented [M11]: luis.coppelli@gmail.com is different from system, please confirm.

The discovery of *Helicobacter pylori* (*H. pylori*) in 1983 started the concept of colonization in the gastric epithelium. For decades, this bacterium has been considered the primary agent responsible for gastric lesions [1,2]. Worldwide, chronic infections caused by *H. pylori* are considered the most extensive [3]. The gastric microbiota was recently described as a new determinant that could affect the epithelium state, which, based on its composition, could also be associated with benign states, chronic inflammation, and even carcinogenesis [4].

Gastric microbiota sequencing studies have reported Firmicutes, Proteobacteria, Actinobacteria, Bacteroidetes, and Fusobacteria phyla colonization [5,6]. This microbiota variability is multifactorial and associated with diet, drugs, antibiotics, immune response, chronic processes, and *H. pylori* infections [7,8].

It has been observed that a chronic *H. pylori* infection can generate physiological changes in the epithelium and the immune system at a gastric level, impacting the state of the gastric microbiota, causing dysbiosis, and favoring the conditions to reach a malignant epithelial state [9–11]. Previous studies indicate that this microbiota can produce inflammation, immunological modifications, reactive oxygen, and nitrogen species, contributing to the development of different lesions, including carcinogenesis [12].

Infection by *H. pylori* can generate a proper environment for the colonization of different bacterial species (unable to survive in the acidic stomach environment), favored by its capacity to produce urease and modify the gastric acidity [13,14]. Consequently, the pH variation reduces the gastric mucus consistency and encourages alternative bacteria to access and colonize the epithelial tissue [15]. However, other studies point out that this new environment generated by *H. pylori* reduces colonizing agents´ diversity, mainly in the cancerous epithelium [6,16]. For these reasons, it is essential to study the interaction between *H. pylori* and gastric microbiota.

In Chile, *H pylori* prevalence reports are found in 44.9% to 78% of infected cases [17,18]. Besides, reports state that the high incidence of gastric cancer is the second national cause of cancer in men (29.7/100.0), the fifth one in women (14.3/100.0), the first cause of deaths in men (25.2/100.0), and the fourth in women (12.5/100.0) [19]. Located in southern Chile, the region of La Araucanía has the fourth-highest amount of reported deaths from gastric cancer in men (36.5/100.0), and the fifth one in women (14.1/100.0) [19]. Besides, the infections produced by *H. pylori* are a risk factor associated with high poverty and low educational levels, along with a high rural population and Mapuche ethnic ascendants in the region [20].

Currently, *H. pylori* infections have been related to gastric dysbiosis, and the alteration in gastric microbiota could be linked with the development of malignant gastric lesions beyond *H. pylori* infection [10,21]. However, in Chile, only a few studies have attempted to describe the gastric microbiota in *H. pylori* infections and its effects on the state of the gastric epithelium [22]. Hence, considering the importance of the possible gastric dysbiosis caused by the *H. pylori* colonization, we proposed to analyze the effect of *H. pylori* colonization on the gastric microbiota and the influence of non-pylori species on the etiology and progression of gastric injuries on the adult population. Then, in this study, we aimed to identify the culturable gastric microbiota and characterize its variability at different stages of the epithelial injury in correspondence with *H. pylori*-infected and non-infected dyspeptic patients from La Araucanía, Chile.

2. Materials and Methods

2.1. Patients and Clinical Specimens

This study includes 155 dyspeptic patients who underwent endoscopy at public (Regional Hospital of Temuco –HHHA- and Villarrica Hospital –HV-) and private (Clinica Alemana of Temuco –CAT-) located southern Chile in the Region of La Araucanía from January 2018 to March 2019.The inclusion criteria of the participants were as follows: patients older than 18 years old, with dyspeptic pathology, who had not taken antibiotics and proton pump inhibitors during three weeks before endoscopy, and voluntarily signed informed consent. The Scientific Ethics Committee of the Universidad de La Frontera, Chile previously approved the informed consent form and the study

Commented [M12]: Please notice that for all commercial material used, please add the information of "manufacture, city, if any state, country"; for all the software used, please add the information of "version, manufacture, city, if any state, country".

Commented [M13]: Should it be em dash?

design (Protocol no. 028/18, date month year). The committee mentioned above also approved this project (code 111_17) by the resolution NO 4183 on October 17th, 2017. Participants were interviewed to complete their information, including age, gender, educational level, Mapuche ethnicity, household, place of residence, smoking and drinking habits, metabolic and cardiovascular diseases (diabetes, hypercholesterolemia, and arterial hypertension), family history of gastric cancer, and *H. pylori* infection history. Every abnormality (gastritis, ulceration, erosion, and others) was recorded during the gastrointestinal endoscopy when examining the esophagus, stomach, and duodenum.

Commented [M14]: Please add the approved date month year.

Two biopsy specimens of the stomach antral zone were obtained from each patient. Antrum samples were chosen as recommended by the standardized biopsies method [19], which the national health institutions used for sampling. Additionally, this sample type is reported as a common stomach area for *H. pylori* colonization and its detection [19]. One biopsy sample was used for the urease test, which was performed in the respective Health Center using the Rapid Urease Test (RUT) CLOtest ® Kimberly Clark, USA. The second sample was stored in a sterile environment in an Eppendorf tube with 200 µL of Brucella Broth (BD-DIFCO, Berkshire, UK) and transported in containers at 4 °C to the Applied Biology Molecular Laboratory of the Centre of Translational Medicine at the Universidad de La Frontera.

2.2. Categorization of State Gastric Epithelial

Based on the endoscopic observation from the gastric mucosa—including the different gastric mucosa (antrum, corpus, fundus) and duodenum—two categories for describing its state were assigned: lesion and non-lesion. Non-lesion indicated tissue without inflammatory or injury signals, while lesion indicated tissue with epithelia showing some gastric or duodenal injuries. Furthermore, the lesion group was subdivided into Non-erosive Lesion (NEL) for the inflammation or non-erosive epithelial injuries, Erosive Lesion (EL) for the tissue with wounds, and Pre-malignant or Malignant Lesion (PML) for gastric atrophic, metaplasia, dysplasia, or gastric cancer [23,24].

Commented [M15]: Should it be em dash?

2.3. Isolation of Culturable Microaerophilic Microbiota

In a period of fewer than two hours after the collection of the samples, the biopsy was macerated and homogenized with sterile polypropylene baguette. A fraction of 100 µL of the homogenate sample was inoculated in Columbia agar (Oxoid CMO 331, UK) enriched with 7% (v/v) equine blood. The other fraction (100 µL) was inoculated in culture media plates (Columbia agar) enriched with 7% (v/v) equine blood and supplemented with the antibiotics Trimethoprim (5 µg mL^{-1}), Amphotericin B (5 µg mL^{-1}), Cefsulodin (5 µg mL^{-1}), and Vancomycin (10 µg mL^{-1}) (Supplement DENT 2%, Oxoid™, Hampshire, UK) to favor the *H. pylori* growth. After seeding, both plates were incubated in a microaerophilic atmosphere (5% O_2, 10% CO_2, 85% N_2, and 90% humidity) using the CampyGen closed system (Oxoid™, Hampshire, UK) from three up to seven days at 37 °C. Visible bacterial colonies were separated according to its phenotype, morphology, Gram stain, catalase, and oxidase test. All isolates were stored at −80 °C in Brucella Broth (BD-DIFCO, Berkshire, UK) with 5% (*v*/*v*) equine serum and 20% glycerol (Sigma-Aldrich, UK) for future molecular identification.

2.4. Identification of Non-pylori Species (Culturable Microaerophilic Microbiota)

Bacterial isolated strains were identified using the MALDI-TOF (Matrix-Assisted-Laser-Desorption-Ionization Time-of-Flight) method with the MALDI-TOF MS Autoflex Speed equipment (Bruker Daltonics, Bremen, Germany) and the Flex Control 1.4 software (Bruker Daltonics, Bremen, Germany), for the automatic acquisition of the mass spectrum in the positive linear mode, in a range of mz^{-1} from 2 to 20 kDa and according to the manufacturer's instructions. The raw spectra were compared using the Biotyper Compass 4.1 database (Bruker Daltonics, Bremen, Germany) for isolated identification. The spectra were recorded at 20 Hz laser frequency, and identification was set from 0 to 3.0, with matching scores higher than 1.7 for genus and 2.0 for species [25].

Bacteria not available in the MALDI TOF database were analyzed by sequencing the *16sRNA* gene. Genomic DNA was obtained from the pure culture using a microbial DNA extraction kit

(DNeasy UltraClean Microbial Kit ® Qiagen, Germany) following the manufacturer's recommendations. Primer 27F 5′-AGAGTTTGATCCTGGCTCAG-3′ and 1392R 5′-GGTTACCTTGTTACGACTT-3′ were used to amplify the conserved region *16sRNA*, as described by Srinivasan et al. [26]. The reaction mixture was initially subjected to a one denaturation cycle at 94 °C for 5 min, followed by 35 cycles: denaturation at 94 °C for 30 s, hybridization at 53 °C for 30 s and extension at 72 °C for 30 s, and finally extension cycle at 72 °C for 5 min in a Labnet digital thermocycler, Multigene Optimax model (LabNet International. Inc). Subsequently, the amplified DNA was visualized in 1% agarose gel. Then, PCR products were purified using the Gel Band purification Kit (GE Healthcare Life Sciences ™, UK) and sequenced in Macrogen Korea®. Sequencing results were analyzed with the MEGA 7.0 program (Molecular Evolutionary Genetic Analysis, Software 64-bit) [27], and compared with the NCBI database (https://www.ncbi.nlm.nih.gov).

Commented [M16]: Please state manufacturer, city and country from where equipment has been sourced.

2.5. Helicobacter pylori Confirmation

Bacteria with suggestive *H. pylori* phenotype were subjected for confirmation using the Polymerase Chain Reaction (PCR). Flanking sequences of the *ureC* gene were amplified using the Fw 5′-AGCTATAAAGTGGGCGAGAG-3′ and Rv 5′ ATTGCACCCGTTAGGCTCAT-3′, as described by Wormwood et al. [28]. A patient was considered infected with *H. pylori* when confirmed positive for the rapid urease test, matching comparison with the *16SrRNA* gene (as established in section 2.4), and when the *ureC* gene was detected from the gastric biopsy.

2.6. Statistical Analyses

Association studies of the characteristics of the patients were carried out using the Graph Pad Prism 8.4.3. program. The Chi-square test of association was used to establish the different characteristics of participants and to compare the diversity and number of isolated species between *H. pylori*-infected and non-infected patients (henceforth referred to as HP+ and HP- groups, respectively) with the different states of the epithelial injury. The comparison of age (average ± SD) between the HP+ and HP- groups were analyzed using t-test. A p-value < 0.05 was considered significant for all the statistical tests.

3. Results

3.1. Characterization and Clinical Data of the Participants

A total of 155 gastric biopsies were obtained from dyspeptic patients during the period of study, 67 (43.2%) were confirmed to be infected with *H. pylori* (HP+ group), and 88 (56.8%) were negative to RUT and did not amplify the *ureC* gen (HP- group). A predominant tendency of women (66.4%, n = 103), Caucasian people (74.2%, n = 115), and residents from urban areas (81.3%, n = 126) was observed. Besides, 29 participants (18.7%) reported previous *H. pylori* eradication therapy, and 11 of them (37.9%) were treatment-resistant. Regarding ethnicity, the Mapuche ethnic participants (n = 40, 25.8%) were mainly HP+ (n = 22, 55.0%) with no significant differences. The others aspects, such as those related to poverty and risk conditions, i.e., rurality (n = 16, 59.3%) and household (n = 28, 59.4%); smoking (n = 27, 67.5) and drinking (n = 41, 57.0%) habits; metabolic disorders, i.e., diabetes (n = 18, 60.0%), hypercholesterolemia (n = 32, 71.1%), arterial hypertension (n = 27, 62.8%) and family history of gastric cancer (n =26, 57.8%) were greater in HP- group, however only hypercholesterolemia showed significant differences (p = 0.032) (Table 1). In general, these results suggest that the patients included in our study have relatively similar lifestyles, excepted for the age and metabolic disease.

Regarding the age range, younger individuals were more significantly represented in HP+, with a mean (± SD) of 48.2± 13.3 years (p = 0.041) (Table 1). *H. pylori* infection' highest prevalence was observed for the 46–55 years-old group (n = 19, 59.4%), which began to decrease significantly after 56 years of age (Figure 1). Furthermore, the 36–45 year-old group had a minor *H. pylori* prevalence than other age ranges (n = 15, 40.5%). It exhibited a more significant population belonging to Mapuche ethnic in HP-.

Table 1. Characteristics of participants according to the *Helicobacter pylori* colonization.

Data	HP+ *n* = 67 *n* (%)	HP- *n* = 88 *n* (%)	Total Participants *n* = 155 *n* (%)	*p* -Value
**Age (years ± *SD*)**	48.2 ± 13.3 [a]	53.4 ± 17 [a]	51.1 ± 15.7 [a]	0.041 *
**Gender**				
Female	44 (42.7)	59 (57.3)	103 (66.4)	0.865
Male	23 (44.2)	29 (55.8)	52 (33.5)	
**Residence**				
Rural	11 (40.7)	16 (59.3)	27 (17.4)	0.833
Urban	55 (43.6)	71 (56.4)	126 (81.3)	
**Ethnicity**				
Mapuche	22 (55.0)	18 (45.0)	40 (25.8)	0.097
Caucasian	45 (39.1)	70 (60.9)	115 (74.2)	
**Other rurality factors**				
Household (≥ *5 members)*	23 (45.1)	28 (54.9)	51 (32.9)	0.863
Education level (≤ 12 years)	37 (41.1)	53 (58.9)	90 (58.1)	0.603
**Addictive habits**				
Smoker	13 (32.5)	27 (67.5)	40 (25.8)	0.139
Drinker (Alcohol)	31 (43.0)	41 (57.0)	72 (46.4)	>0.999
**Metabolic diseases**				
Diabetes	12 (40.0)	18 (60.0)	30 (19.3)	0.838
Hypercholesterolemia	13 (28.9)	32 (71.1)	45 (29.0)	0.032*
**Cardiovascular diseases**				
Arterial hypertension	16 (37.2)	27 (62.8)	43 (27.7)	0.371
**Family history of gastric cancer**	19 (42.2)	26 (57.8)	45 (29.0)	>0.999
***H. pylori* eradication treatment**	11 (37.9)	18 (62.1)	29 (18.7)	0.678

SD = Standard Deviation; * Significant statistical differences $p < 0.05$; a: instead of percentage, age mean values are shown for each group.

Commented [M17]: Please carefully check the accuracy of all data, figures and tables in this manuscript. No changes are allowed after publishing online.

Commented [M18]: Is the bold necessary? Please check throughout the Table.

Commented [M19]: Does a need to be superscripted?

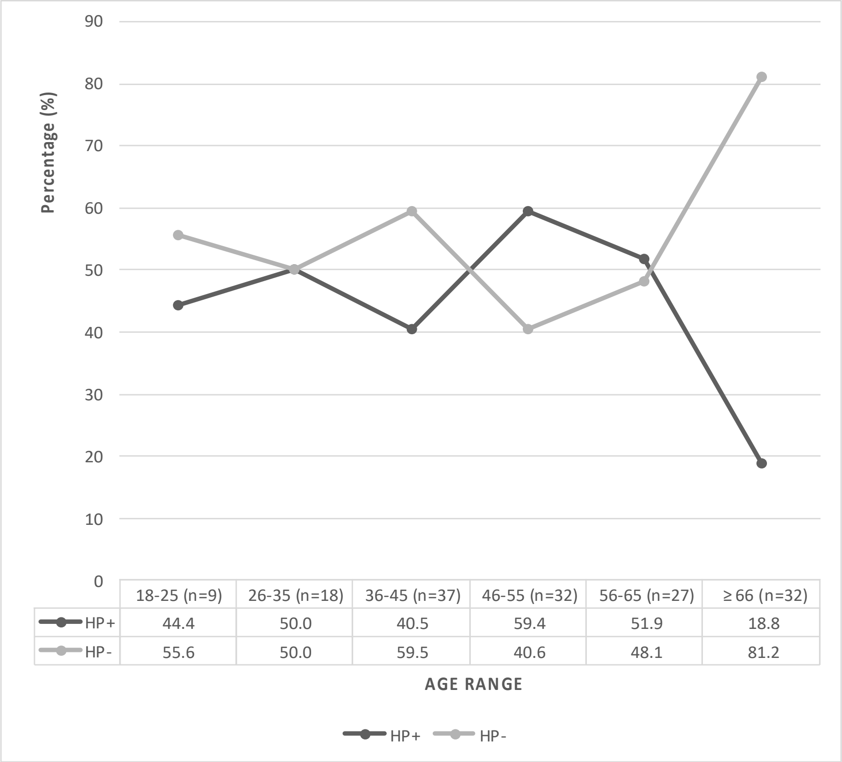

Figure 1. *H. pylori* infection dynamics according to age range. Distribution of patients colonized and not colonized by *H. pylori* according to age and expressed in years.

Table 2. Gastric epithelium state according to injury level and *Helicobacter pylori* colonization.

Gastric Epithelium	HP+ *n* = 67 *n* (%)	HP- *n* = 88 *n* (%)	Total *n* = 155 *n* (%)	*p*-Value
**Non- Lesion**	**47 (45.6)**	**56 (54.4)**	**103 (66.4)**	**0.492**
**Lesion**	**20 (38.5)**	**32 (61.5)**	**52 (33.6)**	**0.772**
▪ Non-erosive lesion (NEL)	11 (35.5)	20 (64.5)	31 (59.6)	0.299
▪ Erosive lesion (EL)	6 (54.5)	5 (45.5)	11 (21.2)	0.722
▪ Premalignant or malignant lesion (PML)	3 (30.0)	7 (70.0)	10 (19.2)	>0.999
- Atrophy	2 (40.0)	3 (60.0)	5 (50.0)	
- Metaplasia	1(20.0)	4 (80.0)	5 (50.0)	

3.2. Bacterial Identification

The microbiological analysis allowed the isolation of bacteria in 126 (81.3%) antrum biopsy samples, corresponding to 48 species, highlighting a wide diversity. Those isolates belonged to the Actinobacteria, Bacteroidetes, Firmicutes, and Proteobacteria phyla, grouped into 18 families and 24 genera (not considering *H. pylori*). The most prevalent non-pylori bacteria were the *Neisseriaceae* (*n*= 33, 21.3%) family, followed by the *Streptococcaceae* (*n* = 31, 20.0%)—with the most extensive diversity of species—and *Actinomycetaceae* (*n* = 14, 9.0%), as well as *Enterobacteriaceae* and *Lactobacillaceae* families, both registered in seven biopsies (4.5% respectively). The remaining bacterial families were

Commented [M20]: Please check if there is Table 2 citation in this manuscript

Commented [M21]: Is there should be Total Participants or Total?

Commented [M22]: Is the bold necessary? Please check throughout the Table.

detected in a minor proportion (Table 3). From all the non-pylori identified families, eight (44.4%) were identified in both groups, and 10 (55.6%) were exclusively present in the non-pylori group (Figure 2). This result corresponded to 42 (89.4%) different species in the HP- and 16 (34.0%) identified on the HP+ group ($p < 0.001$). The main bacterial species found in both groups were *Neisseria flavescens* (*n* = 13, 8.4%), *N. subflava* (*n* = 10, 6.5%), *N. perflava* (*n* = 8, 6.3%), *Streptococcus pneumoniae* (*n* = 9, 5.8%), *S. parasanguinis* (*n*= 8, 5.2%), and *Actinomyces odontolyticus* (*n*= 5, 3.2%). Although *Rothia dentocariosa* (*n* = 6, 3.9%) was one of the most frequent species, it was not detected in the HP+ group (Table 3). It is important to highlight that all these species were more prevalent in the HP- group and that only the *Streptococcaceae* and *Actynomicetaceae* families showed significant differences between both groups ($p < 0.05$) (Figure 2).

Table 3. Culturable bacteria isolated from gastric tissue colonized (HP+) and non-colonized (HP-) by *Helicobacter pylori*.

Nº	Bacteria	Biopsies *n* = 155 *n* (%)	HP+ *n* = 67 *n* (%)	HP- *n* = 88 *n* (%)
**1**	***Neisseriaceae***	**33 (21.3)**	**10 (30.3)**	**23 (69.7)**
1.1	*N. flavescens*	13 (8.4)	5 (38.5)	8 (61.5)
1.2	*N. subflava*	10 (6.5)	2 (20.0)	8 (80.0)
1.3	*N. perflava*	8 (5.2)	2 (25.0)	6 (75.0)
1.4	*N. mucosa*	1 (0.6)	Not detected	1 (100)
1.5	*Eikenella corrodens*	1 (0.6)	1 (100)	Not detected
**2**	***Streptococaceae***	**31 (20.0)**	**6 (19.4)**	**25 (80.6) ***
2.1	*S. pneumoniae*	9 (5.8)	2 (22.2)	7 (77.8)
2.2	*S. parasanguinis*	8 (5.2)	2 (25.0)	6 (75.0
2.3	*S. salivarius*	3 (1.9)	1 (33.3)	2 (66.7)
2.4	*S. anguinosus*	1 (0.6)	Not detected	1 (100)
2.5	*S. mitis*	2 (1.3)	Not detected	2 (100)
2.6	*S. oralis*	2 (1.3)	Not detected	2 (100)
2.7	*S. sanguinis*	2 (1.3)	Not detected	2 (100)
2.8	*S. vestibularis*	1 (0.6)	Not detected	1 (100)
2.9	*S. australis*	1 (0.6)	Not detected	1 (100)
2.10	*S. gordonii*	1 (0.6)	Not detected	1 (100)
2.11	*S. peroris*	1 (0.6)	1 (100)	Not detected
**3**	***Actinomyceteae***	**14 (9.0)**	**1 (7.1)**	**13 (92.8) ***
3.1	*R. dentocariosa*	6 (3.9)	Not detected	6 (100)
3.2	*R. mucilaginosa*	3 (1.9)	Not detected	3 (100)
3.3	*A. odontolyticus*	5 (3.2)	1 (20.0)	4 (80.0)
**4.**	***Enterobacteriaceae***	**7 (4.5)**	**2 (28.6)**	**5 (71.4)**
4.1	*E. coli*	4 (2.6)	1 (25.0)	3 (75.0)
4.2	*E. cloaceae*	1 (0.6)	Not detected	1 (100)
4.3	*K. variicola*	1 (0.6)	Not detected	1 (100)
4.4	*P. penneri*	1 (0.6)	1 (100)	Not detected
**5**	***Lactobacillaceae***	**7 (4.5)**	**2 (28.6)**	**5 (71.4)**
5.1	*L. paracasei*	3 (1.9)	1 (33.3)	2 (66.7)
5.2	*L. agilis*	1 (0.6)	Not detected	1 (100)
5.3	*L. mucosae*	1 (0.6)	Not detected	1 (100)
5.4	*L. rhamnosus*	1 (0.6)	Not detected	1 (100)
5.5	*L. salivarius*	1 (0.6)	1 (100)	Not detected
**6.**	***Gemellaceae***	**3 (1.9)**	**Not detected**	**3 (100)**
6.1	*G. haemolisans*	1 (0.6)	Not detected	1 (100)
6.2	*G. mobillorum*	1 (0.6)	Not detected	1 (100)
6.3	*G. sanguinis*	1 (0.6)	Not detected	1 (100)
**7.**	**B. Non-Fermenters**	**3 (1.9)**	**1 (33.3)**	**2 (66.7)**
7.1	*S. maltophilia*	2 (1.3)	Not detected	2 (100)
7.2	*A. johnsonii*	1 (0.6)	1 (100)	Not detected

Commented [M23]: Is the bold necessary? Please check throughout the Table.

**8**	***Staphylococcaceae***	**3 (1.9)**	**1 (33.3)**	**2 (66.7)**
8.1	*S. pasteuri*	2 (1.3)	1 (50.0)	1 (50.0)
8.2	*S. aureus*	1 (0.6)	Not detected	1 (100)
**9**	***Enterococcaceae***	**2 (1.3)**	**1 (50.0)**	**1 (50.0)**
9.1	*E. faecium*	2 (1.3)	1 (50.0)	1 (50.0)
**10**	***Propionibacteriaceae***	**2 (1.3)**	**Not detected**	**2 (100)**
10.1	*P. acnes*	1 (0.6)	Not detected	1 (100)
10.2	*P. granulosum*	1 (0.6)	Not detected	1 (100)
**11**	***Veillonellaceae***	**2 (1.3)**	**Not detected**	**2 (100)**
11.1	*V. atypical*	1 (0.6)	Not detected	1 (100)
11.2	*V. dispar*	1 (0.6)	Not detected	1 (100)
**12**	***Bacillaceae***	**1 (0.6)**	**Not detected**	**1 (100)**
12.1	*B. cereus*	1 (0.6)	Not detected	1 (100)
**13**	***Carnobacteriaceae***	**1 (0.6)**	**Not detected**	**1 (100)**
13.1	*G. adiacens*	1 (0.6)	Not detected	1 (100)
**14**	***Corynebacteriaceae***	**1 (0.6)**	**Not detected**	**1 (100)**
14.1	*C. glucuronolyticum*	1 (0.6)	Not detected	1 (100)
**15**	***Flavobacteriaceae***	**1 (0.6)**	**Not detected**	**1 (100)**
15.1	*C. sputigena*	1 (0.6)	Not detected	1 (100)
**16**	***Haemophilus***	**1 (0.6)**	**Not detected**	**1 (100)**
16.1	*H. haemolyticus*	1 (0.6)	Not detected	1 (100)
**17**	***Micrococcaceae***	**1 (0.6)**	**Not detected**	**1 (100)**
17.1	*M. luteus*	1 (0.6)	Not detected	1 (100)
**18**	***Prevotellaceae***	**1 (0.6)**	**Not detected**	**1 (100)**
18.1	*P. pallens*	1 (0.6)	Not detected	1 (100)
	**Total bacteria species**	**47 (100)**	**16 (34.0)**	**42 (89.4) ***

B. Non-fermenters = Gram-negative non-fermenting bacilli; *Significant statistical differences $p < 0.05$.

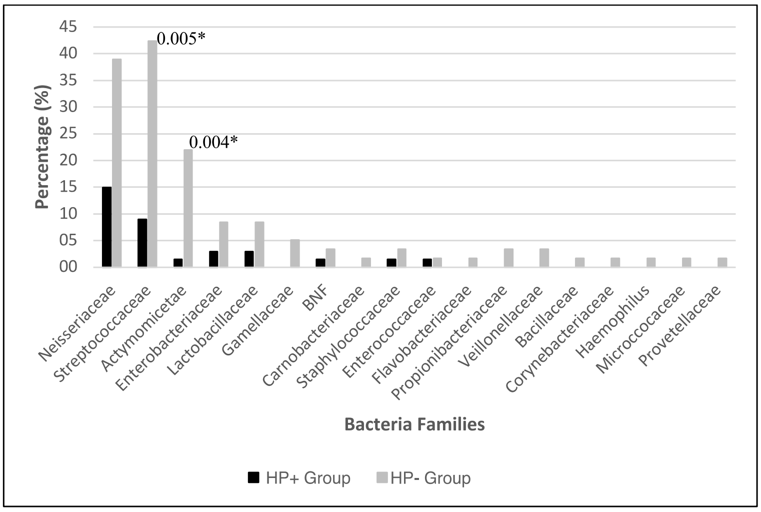

Figure 2. Distribution of cultivable gastric microbiota present in the gastric epithelium. Diversity of cultivable bacterial families isolated from gastric biopsies according to the *H. pylori* (HP+) and non-pylori species colonization (HP-). (BNF: Gram-negative non-fermenting bacilli). *

Commented [M24]: Please give an explanation of the symbol in the Figure.

3.3. Distribution of Culturable Bacteria according to the State of the Gastric Epithelium

Different microbiota profiles (diversity and number of species) were observed according to the epithelium state. An impressive decrease of Gram-negative non-fermenting Bacilli (BNF), (*Haemophilus* and *Flavobacterium)* was detected in the injured epithelium. Those were replaced with different microbiota mainly comprised by Gram-positive bacteria (*Propionibacteriaceae*, *Carnobacteriaceae*, *Micrococcaceae,* and *Prevotellaceae*), representing a potential dysbiosis of the culturable microbiota regarding the epithelial damage progress (Figure 3a,b).

A significant decrease in culturable microbiota diversity was observed in tissues colonized by *H. pylori* (HP+) (Figure 3a). In this group, specifically for the non-erosive lesion, five species belonging to Proteobacteria (*Neisseria*), Actinobacteria (*Actinomyces),* and Firmicutes (*Streptococcus, Lactobacillus, Staphylococcus*) phyla were observed. When the inflamed epithelium progressed towards ulcerous/erosive states, bacterial diversity was reduced to two genera of different phyla, Proteobacteria *(Acinetobacter johnsonii),* and Firmicutes *(Enterococcus faecium)*. Only the Proteobacteria phylum remained in malignant states, specifically the species *Neisseria perflava* and *N. flavescens*. On the other hand, there was a reduced variability of species diversity in the HP- group. In this group, the Proteobacteria, Actinobacteria, Flavobacterium, and Firmicutes phyla were present in all epithelial states except in the advanced damaged ones, in which members of the Flavobacterium phylum were not detected. However, a microbiota dynamic was detected, since it changed from nine genera present on non-erosive lesions (NEL) to five genera on erosive lesions (EL); moreover, it increased to seven genera in malignant lesions (PML) (Figure 3b).

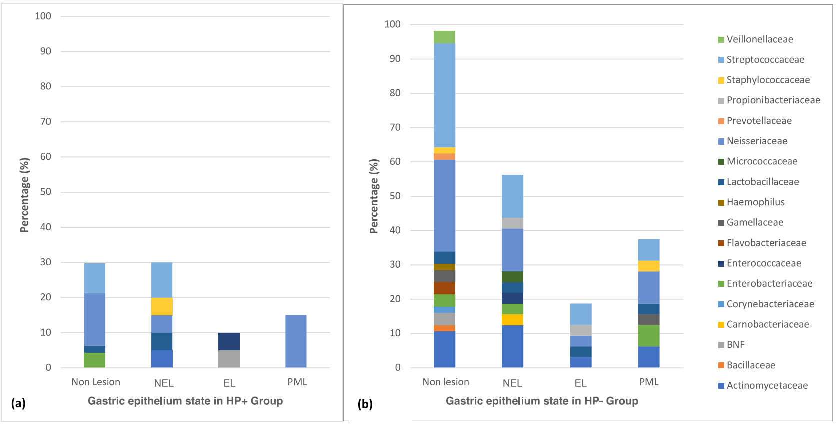

Figure 3. Cultivable gastric microbiota from the gastric epithelium. Distribution of cultivable bacteria present in the different states of the gastric epithelium, according to the HP+ group **(a)** and the HP- group **(b).** (NEL = Non-erosive lesion, EL = Erosive lesion, PML = Premalignant or Malignant lesion (atrophy or metaplasia). BNF: Gram-negative non-fermenting bacilli). BNF: Gram-negative non-fermenting bacilli.

4. Discussion

Despite the harsh conditions of the gastric environment, various studies have described a colonizing microbiota in the stomach. Moreover, it has been recognized that chronic transmissible and non-transmissible diseases can modify this microbiota, affecting the intestinal homeostasis and promoting gastric lesions [29,30]. However, their specific conformation and interaction for disease progression are not fully understood yet. According to our results, bacteria belonging to the Proteobacteria, Firmicutes, Bacteroidetes, and Actinobacteria phyla were identified in the HP- group. Conversely, Proteobacteria, Actinobacteria, and Firmicutes phyla were also identified in the HP+ group showing significantly less abundance (number of species identified from each phylum), even when including participants who had taken an eradication treatment in the HP- group (p = 0.031, Tables S1 and S2). The results mentioned above highlight that bacteria diversity decreases in people infected with *H. pylori*, and might be recovered after eradication treatments, as reported in similar studies [15,31–33].

We found 47 different species distributed in 18 families with a predominance of *Neisseriaceae, Streptococcaceae,* and *Actinomyceteae*, consistent with a Chinese microbiota report regarding dyspeptic patients without an *H. pylori* infection [35]. Moreover, microbiota studies performed in healthy Swedish individuals showed a greater diversity and number of *Streptococcus*, *Actinomyces*, *Prevotella*, and *Gemella* bacteria, specifically in those without an *H. pylori* infection [36]. In this study, those species were also identified in normal and injured epithelia, in both groups, mainly in the *H. pylori* non-infected tissue (HP-).

Commented [M25]: Ref 34 is missing. Please confirm. References must be numbered in order of appearance in the text (including citations in tables and legends) and listed individually at the end of the manuscript.

In the samples colonized by *H. pylori*, less diversity of bacteria and fewer tissues with lesions were detected when compared with the HP- group. This modification in the diversity of bacteria caused by *H. pylori* is consistent with reports that indicate that *H. pylori* causes a change in the colonized environment, reducing the microbiota, mainly in advanced lesions [37]. Probably, once the lesion occurred and its damage and size progressed, the *H. pylori* moved to a healthy epithelium. Besides, due to its mobility, *H. pylori* colonizes throughout the gastric epithelium, it remains in the antral region causing chronic inflammation and histological changes, where lesions can evolve to atrophic gastritis and achlorhydria [6,38]. In turn, advanced lesions might facilitate the growth of other species unable to colonize the acidic environment, while the competition among bacteria promotes coccoid forms of *H. pylori* and finally inhibition of its growth [23,33], driving a gastric

dysbiosis. In our study, *H. pylori* was less frequently detected in pre-malignant lesions (atrophic and metaplasia). Based on the aforementioned preliminary evidence, we cannot ignore the possibility of infected patients where *H. pilory* was localized in other gastric areas but absent in the antral zone. Despite this, it was reported that antral samples yielded similar *H. pylori* detection sensitivity compared with corpus and antrum+corpus samples regarding the atrophic lesion degree [39], which might be necessary for early diagnosis. Therefore, additional studies should be conducted for conclusive results regarding the *H. pylori* sample-dependent detection in infected patients.

Other reports have shown that *H. pylori* can reduce microbiota in children and adults [40]. Also, it has been described that co-excluding interactions between *H. pylori* and *Fusobacterium, Neisseria, Prevotella, Veillonella,* and *Rothia* could be involved in the progression of gastric lesions or carcinogenesis [22,37], while the *H. pylori* eradication treatment allows the microbiota to be restored [22,37,40]. Our results exemplify those observations, showing that *Neisseriaceae, Streptococcaceae, Enterobacteriaceae,* and *Lactobacillaceae* families persist after *H. pylori* infection, while Gram-positive bacteria such as *Gamellaceae, Propionibacteriaceae, Granilucatella, Bacillaceae*, and *Corynebacterium* are excluded even in different states of gastric epithelial injury. Moreover, we showed *Neisseria* species' persistence that were only observed in the atrophic lesions and metaplasia. This decrease in bacterial families also had been reported in gastric biopsies isolates obtained from Chinese dyspeptic patients [9,13,29]. Similarly, Zeng et al. identified *N. flavescens* as a positive urease agent responsible for gastric inflammation and emphasized the importance of the *Neisseria* interaction in the epithelium state [29]; however, studies on its implications are still insufficient. In our research, *Neisseria* was present in erosive tissue and pre-malignant lesions in the HP+ group. Muto et al. stated the carcinogenic capacity of *Neisseria* when cohabiting with *H. pylori* [41]. In that sense, *Neisseria* can produce large amounts of alcohol dehydrogenase, which produces carcinogen acetaldehyde and may also contribute to gastric carcinogenesis [41]. Therefore, *Neisseria* genus' presence should be carefully considered since it was found in advanced lesions of both HP- and HP+ groups, even though Pero et al. indicated that this genus is related to a lower risk of cancer [15]. Beyond the factors above, other studies have pointed out that ancestral population conditions can determine an *H. pylori* infection's benign or malignant course [42]. Recent studies carried out by our research group reported a higher prevalence of *H. pylori* infection in a population with Mapuche ancestry, with a 2.3 times greater risk of being colonized by *H. pylori* [43]. Even though we did not find differences regarding ethnicity, a study focused on Mapuche ethnicity (including a larger number of samples) would be important to investigate the microbiota variability of European and African ancestral *H. pylori* species in the Mapuche population.

The greatest diversity and number of bacteria were observed in tissues non-colonized by *H. pylori* (HP-). From 18 bacterial families detected, only eight genera were recognized on HP+. This microbiota behavior can be attributed to the interaction between the bacterial groups [37], whose detection is often limited because many of these species are not cultivable or impossible to isolate [7]. It should be noticed that other factors such as age, diet, metabolic disorders, smoking and drinking habits can influence the microbiota behavior and how those factors modify the *H. pylori*-associated microbiota should be studied, particularly for age and hypercholesterolemia, which exhibited significant differences in this study.

Moreover, a greater proportion of participants with metabolic diseases and smoking/drinking habits were found in the HP- group—which exhibited the most advanced lesions—suggesting the need for future investigation to relate these parameters with non-*H. pylori*-associated microbiota, as discussed in recent works [44]. Some metabolic disorders can generate a leptin increase and a reduction of vitamins that have been shown to gastric intestinal metaplasia in mice [7] and the modification of human microbiota [8]. Furthermore, these antecedents might favor changes in the gastric epithelium, including the synergistic interaction between the intestinal epithelium, its microbiota, and the immune system, a condition that is considered an essential factor for proper gastrointestinal function [45,46]. Likewise, in our study, patients with a higher prevalence of those factors showed a tendency to more severe lesions of the gastric epithelium.

For erosive tissue colonized by *H. pylori,* we only detected *N. mucosa*, *N. perflava*, *Actinomyces johnsonii,* and *Enterococcus faecium*; however, in pre-malignant lesions, only *N. flavescens* and *N. perflava* remained. *Streptococcus* was detected in all damaged and undamaged tissue stages in the absence of *H. pylori,* and in tissues without lesion and non-erosive lesion, disappearing in the erosive and advanced lesion in the HP+ group. Liu et al., highlighted *Streptococcus* as a vital agent present in the chronic process including gastric cancer, independent of *H. pylori* colonization [21]. Also, Eun et al., detected an increased abundance of the *Streptococcaceae* family in chronic gastritis and intestinal metaplasia of Asian [47]. Here we found that *L. rhamnosus*, *Enterobacteria*, *Rothia*, *Gemella,* and *Neisseria* species remained in injured tissue for HP- and while others *Lactobacillus species* decreased, highlighting the necessity to study these groups in gastric lesions. Other authors have also pointed out a link between *Lactobacillus*/*Streptococcus* species and the progression of lesions towards malignancy [47,48]. In our study, more lesions were found in the HP- group; hence, increasing the samples for better casuistry might be necessary to understand the effect of non-*H. pylori* microbiota on the state of gastric tissue.

Commented [A26]: Please check this sentence as it seems incomplete. Should it say “asian patients”?

The microbiota modifications observed in injured epithelia were mainly associated with an increase in Gram-positive bacteria, specifically from the *Bacillaceae, Carnobacteriaceae, Enterococcaceae, Micrococcaceae,* and *Propionibacteriaceae* families. These species thrive in the oral and respiratory microbiota, suggesting a possible and continued propagation from these anatomical regions to the stomach [15,16,36]. In this sense, a study carried out to characterize transient or resident species in the intestinal microbiota by high-throughput sequencing showed that *H. pylori* influences the oral bacterial community composition, and vice versa [49].

Similar results were also reported for studies carried out with American and Swedish dyspeptic individuals [5,36], which confirmed that an *H. pylori* infection alters the gastric physiology and would be more likely to affect the gastric microbiota [15,31,32,50–52]. Our work confirmed that few culturable species persisted in the malignant epithelium. In gastric cancer of the Swedish population, a decrease of *H. pylori* and an increase in other species such as *Lactobacillus*, *Veillonella, Haemophilus, Streptococcus, Provetella,* and *Neisseria-* was detected [2]. Similar results were reported by Wang et al., who studied the Chinese population with gastric cancer, showing that certain bacteria can produce nitrites and generate inflammation, consequently altering the gastric mucosa [6,15]. Therefore, the interaction and contribution of different bacteria species—such as those reported here—in the epithelium state in HP+ and HP- groups need to be evaluated in future studies. Moreover, new insights are required regarding the microbiota impact on *H. pylori*-associated gastric diseases (e.g., risk of cancer), including its response to treatment and lethality.

5. Conclusions

The culturable gastric microbiota present in the antrum of dyspeptic patients exhibited significant changes associated with an active *H. pylori* infection. We detected a different diversity and abundance of microorganisms directly associated with the different states of the gastric lesion, suggesting that this infection may contribute to the presence of gastric injuries.

The description and identification of culturable microbiota could be the first step for future studies regarding the interaction of bacterial species and the gastric epithelial state, providing an important breakthrough for the understanding of gastric pathologies and, through the microbiota characteristics, defining a bacterial profile to be used as a biomarker for detecting risks in developing of gastric epithelial alterations.

Supplementary Materials: Supplementary materials can be found at www.mdpi.com/xxx/s1, Table S1: Diversity distribution of bacterial Phyla in Pylori and Non-pylori groups including participants without and/or never go on to an *H. pylori* eradication treatment, Table S2: Diversity distribution of bacterial Phyla in Pylori and Non-pylori groups including participants with H. pylori eradication treatment as part of Non/pylori group.

Author Contributions: Conceptualization, C.T., L.B. and M.P.; methodology, C.T., M.O., D.V. and L.C.; validation, A.C., E.H., A.S., and E.R.; formal analysis, C.T. and A.C.; investigation, C.T., M.O. and D.V.; resources, M.P. and L.B.; data curation, A.C., E.H., A.S., and E.R.; writing—original draft preparation, C.T.; writing—review and editing, C.T., M.P. and L.B; visualization, C.T.; supervision, M.P. and L.B.; project

Commented [M27]: Please confirm that every author’s name has been mentioned in this part.

administration, C.T., M.P. and L.B.; funding acquisition, M.P. and L.B. All authors have read and agreed to the published version of the manuscript.

Funding: This work was funded by the NATIONAL COMMISSION OF SCIENCE AND TECHNOLOGY (CONICYT), grant CONICYT–PFCHA/2017–21171513 to C.T.; Fund for the Promotion of Scientific and Technological Development (FONDEF), grant XIII CONCURSO FONIS 2016 SA610197 to M.P; and the RESEARCH NETWORK PROJECT IN EXTREME ENVIRONMENTS (NEXER), grant NXR17–0003 to L.B.

Acknowledgments: The authors wish to thank the Scientific and Technological Bio-resources Nucleus-BIOREN from the Universidad de La Frontera, Chile, for allowing access to the MALDI-TOF equipment. Also, to the endoscopic unit of the Regional Hospital of Temuco, Villarrica Hospital, and Clínica Alemana from Temuco, for facilitating access to dyspeptic patients, their particular gastric biopsy specimens. Besides, we thank Universidad Autónoma de Chile for the grant to support teaching improvement to C.T. Finally, we thank Kattia Núñez-Montero for reviewing and graphical abstract creation (Created with BioRender.com).

Conflicts of Interest: The authors declare no conflict of interest. The funders had no role in the design of the study; in the collection, analyses, or interpretation of data; in the writing of the manuscript, or in the decision to publish the results.

Commented [M28]: Please check and ensure the accuracy of funding names and numbers.

Commented [M29]: We have added this sentence, please check and confirm. Generally, any role of the funders in the design of the study; in the collection, analyses or interpretation of data; in the writing of the manuscript, or in the decision to publish the results must be declared in this section. If there is no role, please state "The funders had no role in the design of the study; in the collection, analyses, or interpretation of data; in the writing of the manuscript, or in the decision to publish the results".

References

1. Marshall, B. WRUnidentified curved bacilli in the stomach of patients with gastritis and peptic ulceration. *Lancet Ltd.* **1984**, *1*, 1311–1314.
2. Dicksved, J.; Lindberg, M.; Rosenquist, M.; Enroth, H.; Jansson, J.K.; Engstrand, L. Molecular characterization of the stomach microbiota in patients with gastric cancer and in controls. *J. Med. Microbiol.* **2009**, *58*, 509–516, doi:10.1099/jmm.0.007302-0.
3. Csendes, A.; Figueroa, M. Situación del cáncer gástrico en el mundo y en Chile. *Rev. Chil. Cirugía* **2016**, *69*, 502–507, doi:10.1016/j.rchic.2016.10.014.
4. Castaño-rodríguez, N.; Goh, K.; Fock, K.M.; Mitchell, H.M.; Kaakoush, N.O. Dysbiosis of the microbiome in gastric carcinogenesis. *Sci. Rep.* **2017**, 1–9, doi:10.1038/s41598-017-16289-2.
5. Bik, E.M.; Eckburg, P.B.; Gill, S.R.; Nelson, K.E.; Purdom, E.A.; Francois, F.; Perez-Perez, G.; Blaser, M.J.; Relman, D.A.Molecular analysis of the bacterial microbiota in the human stomach. *Proc. Natl. Acad. Sci. U. S. A.* **2006**, *103*, 732–737, doi:10.1073/pnas.0506655103.
6. Wang, L.; Zhou, J.; Xin, Y.; Geng, C.; Tian, Z.; Yu, X.; Dong, Q. Bacterial overgrowth and diversification of microbiota in gastric cancer. *Eur. J. Gastroenterol. Hepatol.* **2016**, *28*, 261–266, doi:10.1097/MEG.0000000000000542.
7. Peng, C.; Ouyang, Y.; Lu, N.; Li NThe, N.F.-κ.B. Signaling Pathway, the Microbiota, and Gastrointestinal Tumorigenesis: Recent Advances. *Front. Immunol.* **2020**, *11*, 1–13, doi:10.3389/fimmu.2020.01387.
8. Miftahussurur, M.; Waskito, L.A.; El-Serag, H.B.; Ajami, N.J.; Nusi, I.A.; Syam, A.F.; Matsumoto, T.; Rezkitha, Y.A.A.; Doohan, D.; Fauzia, K.A.; et, al. Gastric microbiota and Helicobacter pylori in Indonesian population. *Helicobacter* **2020**, *25*, 1–10, doi:10.1111/hel.12695.
9. Vogtmann, E.; Goedert, J.J. Epidemiologic studies of the human microbiome and cancer. *Br. J. Cancer* **2016**, *114*, 237–242, doi:10.1038/bjc.2015.465.
10. Khosravi, Y.; Dieye, Y.; Poh, B.H.; Ng, C.G.; Loke, M.F.; Goh, K.L.; Vadivelu, J. Culturable bacterial microbiota of the stomach of helicobacter pylori positive and negative gastric disease patients. *Sci. World, J.* **2014**, *2014*, doi:10.1155/2014/610421.
11. Tang, C.-L. *Helicobacter pylori* tumor necrosis factor-α inducing protein promotes cytokine expression *via* nuclear factor-κB.; *World J. Gastroenterol.* **2013**, *19*, 399, doi:10.3748/wjg.v19.i3.399.
12. Dias-Jácome, E.; Libânio, D.; Borges-Canha, M.; Galaghar, A.; Pimentel-Nunes, P. Gastric microbiota and carcinogenesis: The role of non-Helicobacter pylori bacteria −A systematic review. *Rev. Esp. Enfermedades Dig.* **2016**, *108*, 530–540, doi:10.17235/reed.2016.4261/2016.
13. Das, A.; Pereira, V.; Saxena, S.; Ghosh, T.S.; Anbumani, D. Gastric microbiome of Indian patients with Helicobacter pylori infection , and their interaction networks. *Sci. Rep.* **2017**, 1–9, doi:10.1038/s41598-017-15510-6.
14. Zhang, C.; Cleveland, K.; Schnoll-sussman, F.; Mcclure, B.; Bigg, M.; Thakkar, P.; Schultz, N.; Shah, M.A.; Betel, D. Identification of low abundance microbiome in clinical samples using whole genome sequencing. *Genome Biol.* **2015**, 1–16, doi:10.1186/s13059-015-0821-z.

15. Pero, R.; Brancaccio, M.; Laneri, S.; De Biasi, M.G.; Lombardo, B.; Scudiero, O. A novel view of human Helicobacter pylori infections: Interplay between microbiota and beta-defensins. *Biomolecules* **2019**, *9*, doi:10.3390/biom9060237.
16. Hu, Y.; He, L.H.; Xiao, D.; Liu, G.D.; Gu, Y.X.; Tao, X.X.; Zhang, J.Z. Bacterial flora concurrent with helicobacter pylori in the stomach of patients with upper gastrointestinal diseases. *World, J. Gastroenterol.* **2012**, *18*, 1257–1261, doi:10.3748/wjg.v18.i11.1257.
17. Da Costa, D.; Guidotti, F.; Cabello, N.; Trigo, F.; Contreras, C.; Vergara, F.; Miranda, J.P.; Montenegro, C.; Muñoz, P.; Berger, Z. Disminución en la frecuencia de infección por Helicobacter pylori en endoscopías digestivas altas. *Rev. Med. Chil.* **2018**, *146*, 555–561, doi:10.4067/s0034-98872018000500555.
18. González, I.; Romero, J.; Rodríuez, B.; Llanos, J.; Morales, E.; Figueroa, H.; Perez-Castro, R.; Valdés, E.; Cofre, C.; Rojas, A. High prevalence of virulence-associated genotypes in Helicobacter pylori clinical isolates in the Region del Maule, Chile. *Scand. J. Infect. Dis.* **2011**, *43*, 652–655, doi:10.3109/00365548.2011.572909.
19. Minsal Plan Nacional d.e. Cancer 2018-2028. Documento de consulta pública. *Minist. Salud, Chile, Minsal 2018*. Available online: *https//www.gob.cl/plannacionaldecancer/***2018**, 1–27 (accessed on).
20. Ministerio de Desarrollo Social Informe de Desarrollo Social 2017 (informe en proceso de edición). **2017**. Available online: (accessed on).
21. Liu, J.; Xue, Y.; Zhou, L. Detection of gastritis-associated pathogens by culturing of gastric juice and mucosa. *Int. J. Clin. Exp. Pathol.* **2018**, *11*, 2214–2220.
22. Brawner, K.M.; Kumar, R.; Serrano, C.A.; Ptacek, T.; Lefkowitz, E.; Morrow, C.D.; Zhi, D.; Kyanam-Kabir-Baig, K.R.; Smythies, L.E.; Harris, P.R.; et al. Helicobacter pylori infection is associated with an altered gastric microbiota in children. *Mucosal Immunol.* **2017**, *10*, 1169–1177, doi:10.1038/mi.2016.131.
23. Sotelo, S.; Manterola, C. Repercusiones Diagnóstico-Terapéuticas de las Lesiones Preneoplásicas Gástricas. *Int. J. Morphol.* **2019**, *37*, 917–927, doi:10.4067/s0717-95022019000300917.
24. Correa, P.; Piazuelo, M.B. The gastric precancerous cascade. *jJ Dig Dis* **2012**, *13*, 2–9, doi:10.1111/j.1751-2980.2011.00550.x.The.
25. García-Rodríguez, J.; Quiles-Melero, I.; Gómez-López, A.; Mingorance, J. Evaluation of matrix-assisted laser desorption/ionisation time-of-flight (MALDI-TOF) mass spectrometry for identification of Candida parapsilosis, C. orthopsilosis and C. metapsilosis. *Eur. J. Clin. Microbiol. Infect. Dis.* **2012**, *31*, 67–71, doi:10.1007/s10096-011-1277-z.
26. Srinivasan, R.; Karaoz, U.; Volegova, M.; MacKichan, J.; Kato-Maeda, M.; Miller, S.; Nadarajan, R.; Brodie, E.L.; Lynch, S.V. Use of 16S rRNA gene for identification of a broad range of clinically relevant bacterial pathogens. *PLoS ONE* **2015**, *10*, 1–22, doi:10.1371/journal.pone.0117617.
27. Kumar, S.; Stecher, G.; Tamura, K. MEGA7: Molecular Evolutionary Genetics Analysis Version 7.0 for Bigger Datasets. *Mol. Biol. Evol.* **2016**, *33*, 1870–1874, doi:10.1093/molbev/msw054.
28. Wormwood, T.; Parra, A.; Bresky, G.; Madariaga, J.A.; Sergio, H.; Jacqueline, F.; Bernal, G. Prevalencia de cepas cagA-positivo en la región de Coquimbo, determinada mediante nested-qPCR en muestras fecales. *Rev. Med. Chil.* **2018**, *146*, 596–602.
29. Zeng, B.; Sun, L.; Chen, Y.; Qian, Y.; Cao, Q.; Zhang, Z.; Li, Z. Neisseria flavescens: A Urease-Expressing Potential Pathogen Isolated from Gastritis Patients. *Curr. Microbiol.* **2018**, *75*, 186–193, doi:10.1007/s00284-017-1364-1.
30. Gupta, V.K.; Paul, S.; Dutta, C. Geography, ethnicity or subsistence-specific variations in human microbiome composition and diversity. *Front. Microbiol.* **2017**, *8*, doi:10.3389/fmicb.2017.01162.
31. Yang, I.; Woltemate, S.; Piazuelo, M.B.; Bravo, L.E.; Yepez, M.C.; Romero-Gallo, J.; Delgado, A.G.; Wilson, K.T.; Peek, R.M.; Correa, P.; et al. Different gastric microbiota compositions in two human populations with high and low gastric cancer risk in Colombia. *Sci. Rep.* **2016**, *6*, 1–10, doi:10.1038/srep18594.
32. Wang, D.; Li, Y.; Zhong, H.; Ding, Q.; Lin, Y.; Tang, S.; Zong, Y.; Wang, Q.; Zhang, X.; Yang, H.; et al. Alterations in the human gut microbiome associated with Helicobacter pylori infection. *FEBS Open Bio* **2019**, *9*, 1552–1560, doi:10.1002/2211-5463.12694.
33. Ianiro, G.; Molina-infante, J.; Gasbarrini, A. Gastric Microbiota. *Helicobacter ISSN 1523-5378* **2015**, *20*, 68–71, doi:10.1111/hel.12260.
34. Jayaraman, A.; Wood, T.K. Bacterial Quorum Sensing: Signals, Circuits, and Implications for Biofilms and Disease. *Annu. Rev. Biomed. Eng.* **2008**, *10*, 145–167, doi:10.1146/annurev.bioeng.10.061807.160536.

Commented [M30]: Please provide the exact date when you last accessed the link.

Commented [M31]: Please cite the website as a reference in the following format: Title. Available online: http://www.alz.org/what-is-dementia.asp (accessed on Day Month Year).

Commented [M32]: Please add the website.

Commented [M33]: Please provide the exact date when you last accessed the link.

Commented [M34]: Please confirm the title of reference, make it match the doi.

Commented [M35]: Please check if ref 34 is cited in Main Text.

35. Li, X.X.; Wong, G.L.H.; To, K.F.; Wong, V.W.S.; Lai, L.H.; Chow, D.K.L.; Lau, J.Y.W.; Sung, J.J.Y.; Ding, C. Bacterial microbiota profiling in gastritis without Helicobacter pylori infection or non-steroidal anti-inflammatory drug use. *PLoS ONE* **2009**, *4*, 1–9, doi:10.1371/journal.pone.0007985.
36. Andersson, A.F.; Lindberg, M.; Jakobsson, H.; Ba, F. Comparative Analysis of Human Gut Microbiota by Barcoded Pyrosequencing. *PLoS ONE* **2008**, *3*, doi:10.1371/journal.pone.0002836.
37. Guo, Y.; Zhang, Y.; Gerhard, M.; Gao, J.J.; Mejias-Luque, R.; Zhang, L.; Vieth, M.; Ma, J.L.; Bajbouj, M.; Suchanek, S.; et al. Effect of Helicobacter pylori on gastrointestinal microbiota: A population-based study in Linqu, a high-risk area of gastric cancer. *Gut* **2019**, *69*, 1598–1607, doi:10.1136/gutjnl-2019-319696.
38. Ailloud, F.; Didelot, X.; Woltemate, S.; Pfaffinger, G.; Overmann, J.; Bader, R.C.; Schulz, C.; Malfertheiner, P.; Suerbaum, S. Within-host evolution of Helicobacter pylori shaped by niche-specific adaptation, intragastric migrations and selective sweeps. *Nat. Commun.* **2019**, doi:10.1038/s41467-019-10050-1.
39. Lan, H.C.; Chen, T.S.; Li, A.F.Y.; Chang, F.Y.; Lin, H.C. Additional corpus biopsy enhances the detection of Helicobacter pylori infection in a background of gastritis with atrophy. *BMC Gastroenterol.* **2012**, doi:10.1186/1471-230X-12-182.
40. Serrano, C.A.; Pierre, R.; Van Der Pol, W.J.; Morrow, C.D.; Smith, P.D.; Harris, P.R. Eradication of Helicobacter pylori in Children Restores the Structure of the Gastric Bacterial Community to That of Noninfected Children. *Gastroenterology* **2019**, *157*, 1673–1675, doi:10.1053/j.gastro.2019.08.017.
41. Muto, M.; Hitomi, Y.; Ohtsu, A.; Shimada, H.; Kashiwase, Y.; Sasaki, H.; Yoshida, S.; Esumi, H. Acetaldehyde production by non-pathogenic Neisseria in human oral microflora: Implications for carcinogenesis in upper aerodigestive tract. *Int. J. Cancer* **2000**, *88*, 342–350, doi:10.1002/1097-0215(20001101)88:3<342::AID-IJC4>3.0.CO;2-I.
42. Thorell, K.; Yahara, K.; Berthenet, E.; Lawson, D.J.; Mikhail, J.; Kato, I.; Mendez, A.; Rizzato, C.; Bravo, M.M.; Suzuki, R.; et al. Rapid evolution of distinct Helicobacter pylori subpopulations in the Americas. *PLoS Genet.* **2017**, *13*, doi:10.1371/journal.pgen.1006546.
43. Oporto, M.; Pavez, M.; Troncoso, C.; Cerda, A.; Hofmann, E.; Sierralta, A.; Rios, E.; Coppelli, L.; Barrientos, L. Prevalence of Infection and Antibiotic Susceptibility of Helicobacter pylori : An Evaluation in Public and Private Health Systems of Southern Chile. *Pathogens* **2019**, *8*, 1–12, doi:10.3390/pathogens8040226.
44. Ndegwa, N.; Ploner, A.; Andersson, A.F.; Zagai, U.; Andreasson, A.; Vieth, M.; Talley, N.J.; Agreus, L.; Ye, W. Gastric Microbiota in a Low-Helicobacter pylori Prevalence General Population and Their Associations With Gastric Lesions. *Clin. Transl. Gastroenterol.* **2020**, *11*, e00191, doi:10.14309/ctg.0000000000000191.
45. Weng, M.T.; Chiu, Y.T.; Wei, P.Y.; Chiang, C.W.; Fang, H.L.; Wei, S.C. Microbiota and gastrointestinal cancer. *J. Formos. Med. Assoc.* **2019**, *118*, S32–S41, doi:10.1016/j.jfma.2019.01.002.
46. Diehl, G.E.; Longman, R.S.; Zhang, J.X.; Breart, B.; Galan, C.; Cuesta, A.; Schwab, S.R.; Littman, D.R. Microbiota restricts trafficking of bacteria to mesenteric lymph nodes by CX 3 CR1 hi cells. *Nature* **2013**, *494*, 116–120, doi:10.1038/nature11809.
47. Eun, C.S.; Kim, B.K.; Han, D.S.; Kim, S.Y.; Kim, K.M.; Choi, B.Y.; Song, K.S.; Kim, Y.S.; Kim, J.F. Differences in gastric mucosal microbiota profiling in patients with chronic gastritis, intestinal metaplasia, and gastric cancer using pyrosequencing methods. *Helicobacter* **2014**, *19*, 407–416, doi:10.1111/hel.12145.
48. Gantuya, B.; El Serag, H.B.; Matsumoto, T.; Ajami, N.J.; Uchida, T.; Oyuntsetseg, K.; Bolor, D.; Yamaoka, Y. Gastric mucosal microbiota in a Mongolian population with gastric cancer and precursor conditions. *Aliment. Pharmacol. Ther.* **2020**, *51*, 770–780, doi:10.1111/apt.15675.
49. Schulz, C.; Schütte, K.; Koch, N.; Vilchez-Vargas, R.; Wos-Oxley, M.L.; Oxley, A.P.A.; Vital, M.; Malfertheiner, P.; Pieper, D.H. The active bacterial assemblages of the upper Gi tract in individuals with and without Helicobacter infection. *Gut* **2016**, *67*, doi:10.1136/gutjnl–2016–312904.
50. Ge, Z.; Sheh, A.; Feng, Y.; Muthupalani, S.; Ge, L.; Wang, C.; Kurnick, S.; Mannion, A.; Whary, M.T.; Fox, J.G. Helicobacter pylori-infected C57BL/6 mice with different gastrointestinal microbiota have contrasting gastric pathology, microbial and host immune responses. *Sci. Rep.* **2018**, *8*, 1–15, doi:10.1038/s41598-018-25927-2.
51. Kienesberger, S.; Cox, L.M.; Livanos, A.; Zhang, X.S.; Chung, J.; Perez-Perez, G.I.; Gorkiewicz, G.; Zechner, E.L.; Blaser, M.J. Gastric Helicobacter pylori Infection Affects Local and Distant Microbial Populations and Host Responses. *Cell Rep.* **2016**, *14*, 1395–1407, doi:10.1016/j.celrep.2016.01.017.
52. Xia, Y.; Meng, G.; Zhang, Q.; Liu, L.; Wu, H.; Shi, H.; Bao, X.; Su, Q. Dietary Patterns are Associated with Helicobacter Pylori Infection in Chinese Adults : A Cross-Sectional Study. *Sci. Rep.* **2016**, 1–8, doi:10.1038/srep32334.

© 2020 by the authors. Licensee MDPI, Basel, Switzerland. This article is an open access article distributed under the terms and conditions of the Creative Commons Attribution (CC BY) license (http://creativecommons.org/licenses/by/4.0/).
